# Attitudes and Readiness of Students of Healthcare Professions towards Interprofessional Learning

**DOI:** 10.1371/journal.pone.0168863

**Published:** 2017-01-06

**Authors:** Mari Kannan Maharajan, Kingston Rajiah, Suan Phaik Khoo, Dinesh Kumar Chellappan, Ranjit De Alwis, Hui Cing Chui, Lui Lee Tan, Yee Ning Tan, Shin Yee Lau

**Affiliations:** 1 Department of Pharmacy Practice, School of Pharmacy, International Medical University, Kuala Lumpur, Malaysia; 2 Division of Oral Diagnostic Sciences, School of Dentistry, International Medical University, Kuala Lumpur, Malaysia; 3 Department of Life Sciences, School of Pharmacy, International Medical University, Kuala Lumpur, Malaysia; 4 Department of Community Medicine, School of Medicine, International Medical University, Kuala Lumpur, Malaysia; Kyoto University, JAPAN

## Abstract

**Objectives:**

To evaluate the attitudes and readiness of students of healthcare professions towards interprofessional learning.

**Methodology:**

A cross-sectional study design was used. Two different scales were used to measure the readiness for and perception of interprofessional learning; these were the 'Readiness for Interprofessional Learning Scale' and the 'Interdisciplinary Education Perception Scale'. A convenience sampling method was employed. The sample was drawn from undergraduate students enrolled in years 1 to 5 of medical, dental, pharmacy and health sciences programme. Descriptive and inferential statistics were used to analyse the data.

**Results:**

The overall response rate was 83%. The students mentioned that shared learning with other healthcare professional students will increase their ability to understand clinical problems. The students also mentioned that such shared learning will help them to communicate better with patients and other professionals. The students preferred to work with individuals from their own profession. Participants from medical, dental, pharmacy, and health sciences had a difference in opinion about 'negative professional identity', a domain of the Readiness for Interprofessional Learning Scale. Based on the different year of study of the students, 'team work and collaboration', 'negative professional identity' and 'roles and responsibility' were the Interdisciplinary Education Perception Scale domains where students had a difference in opinion.

**Conclusions:**

Attitudes and readiness towards interprofessional learning showed significant differences among students of various healthcare professions; these differences also depended on the students' year of study. Interprofessional learning should be incorporated in the curriculum of all healthcare professional programs, which may foster students to become competent healthcare providers and understand each profession's role.

## Introduction

Effective interprofessional learning (IPL) plays a major role in preparing individual health professional students for future collaborative healthcare practice. A multi-disciplinary healthcare team can provide better treatment outcomes and quality of care compared to that delivered by a team of individuals from a single discipline [1, 2]. In an interprofessional team, the professionals from different disciplines each make unique contributions to achieving a better quality of life and improved safety for their patients [3]. The World Health Organization (WHO) considers that interdisciplinary collaborative practice in healthcare settings strengthens patient management frameworks. Furthermore, it regards IPL as one of the most promising strategies to achieve effective collaborative practice in healthcare settings [4]. IPL helps students to be familiar with the key roles of their respective jobs in addition to the roles of their team members from other professions.

Recent studies suggested that the value of prior experience with IPL in the workplace lies in improved professional identities and attitudes towards teamwork [5–8]. At the present time, the didactic educational component of each professional course is specific to that discipline with minimal interdisciplinary exposure until students enter clinical training [9]. Given that preliminary evidence indicates that interprofessional care improves healthcare outcomes, it seems that traditional professional programmes would benefit from implementation of structured IPL experiences. These would provide the necessary prior exposure needed for health professional students to shape their professional attitudes and competencies for future interprofessional collaboration. In particular, early interactions among students from different disciplines during IPL could help them to understand the importance of interpersonal skills required for communication in the healthcare workplace, which in turn may reduce clinical procedural errors that might endanger patients [10–12]. It has been shown that medical errors can be reduced through improved interdisciplinary communication, which is one of the benefits of IPL, particularly when the learning groups have balanced input from each of the other professions [11, 12]. IPL participation by all health professional students should also be coordinated across different disciplines, in order to enable a better IPL experience [13].

However, health professional students from discipline-focussed programmes may have diverse attitudes and readiness towards participation in the IPL. The students’ preparedness to engage in IPL will be directly dependent on their attitudes and readiness [14–16]. Positive attitudes towards IPL and readiness for IPL could favour the outcomes of interprofessional collaboration. Students’ attitudes and acceptance of interprofessional collaborative practice may be influenced importantly by their willingness to participate in IPL. Therefore, evaluating students’ attitudes and readiness for IPL would provide an insight into their willingness and preparedness for meaningful interprofessional collaboration. This study aimed to evaluate the attitudes of undergraduate healthcare professional students towards IPL and their readiness to participate in IPL activities.

## Methodology

### Study design

A cross-sectional study was conducted for the period of 6 months from June to November, 2015 in the International Medical University, Malaysia. Convenience sampling was undertaken. The sample size was calculated using Raosoft software by considering the total population of students during the study period; power was kept at 80%; response distribution as 50%; confidence interval was set at 95 and margin of error was set as 5%. All the students were invited to participate in the study. The minimum sample size required to generalize the findings, were 175 medical students, 153 pharmacy students, 76 dentistry students, and 168 health sciences students. However, 232 medical students, 223 pharmacy students, 77 dentistry students and 277 health sciences students completed the survey, which was more than the minimum required sample size. Students who were not interested in participating were excluded from this study.

### Setting, participants and ethical considerations

The study was conducted at the International Medical University, Kuala Lumpur, Malaysia which provides healthcare related programs. All the undergraduate programs have embedded IPL in their curricula. The sample was drawn from all undergraduate students enrolled in years 1 to 5 of the disciplines listed above. Participation was voluntary. A written consent was obtained from the students prior to receiving the questionnaire. This study protocol was accepted by the International Medical University-Joint Committee for Ethics and Research of (BP I-01-12 (30) 2015).

### Study instruments

The Readiness for Interprofessional Learning Scale (RIPLS), which enabled the students to reflect on various aspects of IPL, was used to measure student readiness, or student beliefs, about IPL. A 5-point Likert Scale (Strongly agree = 5, agree = 4, neutral = 3, disagree = 2 and strongly disagree = 1) was used to analyse the students’ responses [17]. The study tool has 19 self-reported items under four different domains. Domain 1 focused on the aspects of teamwork and collaboration (item 1–9). Domain 2 focused on negative professional identity towards other professions (item 10–12). The items in this domain were negatively worded. Therefore, these items were reverse-scored to calculate the overall mean score, i.e., strongly disagree = 5, disagree = 4, neutral = 3, agree = 2 and strongly agree = 1. Domain 3 focused on positive professional identity (item 13–16). Domain 4 focused on the roles and responsibilities of professionals (item 17–19). A higher mean score represents a positive attitude towards IPL. The intra-class correlation of this scale was 0.76 and the Cronbach’s α was 0.90 [15, 17, 18].

The Interdisciplinary Education Perception Scale (IEPS) was the second instrument used in the study to detect changes in learning over time among health professional students. It consisted of three domains (Competency and Autonomy, Perceived Need for Cooperation, and Perception of Actual Cooperation) with 12 items. The validated instrument used a 6-point Likert-scale (Strongly disagree = 1, moderately disagree = 2, somewhat disagree = 3, somewhat agree = 4, moderately agree = 5 and strongly agree = 6). The scale has a Cronbach’s alpha of 0.80 with a test reliability of 0.60. A higher mean score represents positive attitudes towards IPL.

Additionally, the participants’ demographic details (age, gender, ethnicity, programme of study and prior experience of IPL) were also collected.

### Data analysis

Statistical Package for the Social Sciences (SPSS) Version 18.0, SPSS Inc., Chicago, Illinois, U.S.A. was used to analyse the data. The demographic data of the respondents were analysed and described in terms of means (M) and standard deviations (SD). The rest of the data had been analysed using one-way ANOVA test. Significant differences were analysed using Tukey’s post hoc test. The results were considered to be statistically significant where p was < 0.05.

## Results

### Respondents’ demographics

Of the total student population of 975, 809 responded, making an overall response rate of 83%. Respondents were from undergraduate programmes in health sciences (n = 277) medicine (n = 232), pharmacy (n = 223) and dentistry (n = 77) undergraduate programmes. The details of respondents’ demography are shown in Table 1.

**Table 1:** Respondents’ demographic details.

Variable	Descriptor	n (%)
**Gender **	Male	262 (32.39)
	Female	547 (67.61)
**Year of education**	Year 1	135 (16.69)
	Year 2	162 (20.02)
	Year 3	219 (27.07)
	Year 4	237 (29.30)
	Year 5	56 (6.92)
**Ethnicity **	Malay	160 (19.78)
	Chinese	446 (55.13)
	Indian	183 (22.62)
	Others	20 (2.47)
**Prior exposure to IPE **	Yes	331 (40.91)
	No	478 (59.08)
**Programme of study**	Health sciences	277 (34.24)
**(Discipline)**	Medicine	232 (28.68)
	Pharmacy	223 (27.56)
	Dentistry	77 (9.52)

The response rates for different disciplines were 94% (277/295) from health sciences, 89% (223/251) from pharmacy, 72% (232/320) from medicine, and 71% (77/109) from dentistry. The response rates for different year of the study were 75% (135/180) from year 1, 81% (162/199) from year 2, 83% (219/262) from year 3, 86% (237/274) from year 4 and 93% (56/60) from the year 5.

### The Readiness for Interprofessional Learning Scale (RIPLS)

The mean scores of each item of RIPLS are shown in Table 2. The highest mean score was obtained for the statement ‘*Shared learning with other healthcare professional students will increase their ability to understand clinical problems’*. The second highest mean score was obtained for the statement ‘*Shared learning with other healthcare professionals will help me to communicate better with patients and other professionals’*. Students obtained lowest mean score, for the statement ‘*I have to acquire much more knowledge and skill than other students*’ and ‘I *am not sure what my professional role will be’*.

**Table 2:** Mean scores of each item of RIPLS (n = 809).

**Item**	**Mean**	**(SD)**
**Domain 1: Teamwork and collaboration**		
*1.* ***Learning with other students will make me a more effective member of a healthcare team***	4.29	(0.59)
*2.* ***Patients would ultimately benefit if healthcare students worked together***	4.25	(0.60)
*3.* ***Shared learning with other healthcare students will increase my ability to understand clinical problems***	4.38	(0.56)
*4.* ***Communication skills should be learned withy other healthcare students***	4.15	(0.69)
*5.* ***Team-working skills are vital for all healthcare students to learn***	4.20	(0.61)
*6.* ***Shared learning will help me to understand my own professional limitations***	4.23	(0.58)
*7.* ***Learning between healthcare students before qualification would improve working relationships after qualification***	4.16	(0.72)
*8.* ***Shared learning will help me think positively about other healthcare professionals***	4.08	(0.74)
*9.* ***For small-group learning to work, students need to respect and trust each other***	4.21	(0.58)
**Domain 2: Negative professional identity**		
*10.* I don't want to waste time learning with other healthcare students*, ^Ϯ^	3.96	(0.88)
*11.* It is not necessary for undergraduate healthcare students to learn together*, ^Ϯ^	3.84	(0.80)
*12.* Clinical problem solving can only be learnt effectively with students from my own school*, ^Ϯ^	3.91	(0.97)
**Domain 3: Positive professional identity**		
*13.* ***Shared learning with other healthcare professionals will help me to communicate better with patients and other professionals***	4.31	(0.79)
*14.* ***I would welcome the opportunity to work on small group projects with other healthcare students***	4.25	(0.54)
*15.* ***Shared learning will help me clarify the nature of patients' or clients' problems***	4.28	(0.73)
*16.* ***Shared learning before qualification will help me become a better team worker***	4.24	(0.63)
**Domain 4: Roles and responsibilities**		
*17.* ***The function of allied health professionals are mainly to provide support for doctors***	3.74	(0.91)
*18.* ***I am not sure what my professional role will be***	3.54	(0.93)
*19.* ***I have to acquire much more knowledge and skill than other students***	3.54	(1.02)

* Negatively worded item.

^Ϯ^ Negatively worded items were reverse-scored to calculate the overall mean score.

### The Interdisciplinary Education Perception Scale (IEPS)

The mean scores of each item of IEPS in detail are shown in Table 3. The highest mean score was obtained for the statement *‘Individuals in my profession work well with each other’*. The second highest mean score was obtained for the statement *‘Individuals in my profession are willing to share information and resources with other professionals*’. The lowest mean score was obtained for the statement *Individuals in my profession must depend upon the work of people in other professions*.

**Table 3:** Mean scores of each item of IEPS (n = 809).

Item	Mean	(SD)
**Domain 1: Competency & Autonomy**		
*1.* ***Individuals in my profession are well-trained***	4.12	(0.64)
*2.* ***Individuals in my profession are very positive about their goals and objectives***	4.18	(0.58)
*3.* ***Individuals in my profession are very positive about their contributions and accomplishments***	4.12	(0.56)
*4.* ***Individuals in my profession trust each other's professional judgement***	4.09	(0.73)
*5.* ***Individuals in my profession are extremely competent***	3.96	(0.72)
**Domain 2: Perceived Need for Cooperation**		
*6.* ***Individuals in my profession need to cooperate with other professions***	4.11	(0.78)
*7.* ***Individuals in my profession must depend upon the work of people in other professions***	3.66	(0.98)
**Domain 3: Perception of actual cooperation**		
*8.* ***Individuals in my profession are able to work closely with individuals in other professions***	4.01	(0.68)
***9. Individuals in my profession are willing to share information and resources with other professionals***	4.18	(0.58)
*10.* ***Individuals in my profession have good relations with people in other professions***	3.96	(0.78)
*11.* ***Individuals in my profession think highly of other related professions***	4.12	(0.74)
*12.* ***Individuals in my profession work well with each other***	4.21	(0.68)

### Domain comparisons

Table 4 shows the students’ RIPLS and IEPS domain scores for the various disciplines. Participants from dental, medical, pharmacy, and health sciences showed differences in opinion about ‘negative professional identity’, a domain of the RIPLS scale. The students from medicine scored significantly higher than the students from all other disciplines (F (3,805) = 6. 92; p = 0. 041). The other RIPLS domains such as ‘teamwork and collaboration’, ‘positive professional identity’ and ‘roles and responsibilities’ did not show any significant difference among students from different disciplines.

**Table 4:** RIPLS and IEPS domain scores for each discipline (mean and standard deviation).

**Domain**	**Dentistry Mean (SD)**	**Medicine Mean (SD)**	**Pharmacy Mean (SD)**	**Health Sciences Mean (SD)**	**F- ratio**
**RIPLS**	**76.85 (2.29)**	**75.08 (2.69)**	**77.44 (2.17)**	**76.88 (2.83)**	
Teamwork and collaboration	38.00 (3.74)	38.62 (3.67)	38.49 (3.57)	39.13 (4.03)	F(3,805) = 2.74 p = 0.064
Negative professional identity	11.55 (2.26)	11.11 (2.70)	11.91 (1.68)	10.74 (3.06)	F(3,805) = 6.92 p = 0.041
Positive professional identity	16.57 (1.66)	16.52 (2.31)	16.49 (1.78)	16.57 (2.52)	F(3,805) = 2.03 p = 0.253
Roles and responsibilities	10.73 (1.48)	8.83 (2.08)	10.55 (1.65)	10.44 (1.69)	F(3,805) = 2.65 p = 0.067
**IEPS**	**47.76 (1.84)**	**48.83 (1.59)**	**44.41 (2.06)**	**47.43 (2.7)**	
Competency and autonomy	20.14 (2.14)	20.59 (1.85)	18.35 (2.06)	19.71 (2.94)	F(3,805) = 2.75 p = 0.077
Perceived need for cooperation	7.48 (1.22)	8.10 (1.04)	7.06 (1.12)	7.75 (1.32)	F(3,805) = 1.05 p = 0.506
Perception of actual cooperation	20.14 (2.17)	20.14 (1.89)	19.00 (3.01)	19.97 (2.84)	F(3,805) = 2.25 p = 0.027

Likewise, the students had a difference in opinion about the IEPS domain ‘competency and autonomy’. The scores of medical students were significantly higher than those of students from other disciplines (F (3,805) = 2. 75; p = 0. 037). The other domains of IEPS such as ‘perceived need for cooperation’ and ‘perception of actual cooperation’ did not have show significant differences among the students.

Table 5 shows the RIPLS and IEPS domain scores for different year of study. Based on the students’ different year of study, ‘team work and collaboration’, ‘negative professional identity’, and ‘roles and responsibility’ were the IEPS domains where students had a difference in opinion. The students from years 2 and 4 had a difference in opinion about the ‘team work and collaboration’. The year 4 students scored significantly higher than the year 2 students (F (4,804) = 4. 45; p = 0. 001). Likewise, the year 2 students and year 4 students showed a difference in opinion about ‘negative professional identity’. The year 4 students had significantly lower scores than the year 2 students (F (4,804) = 6. 43; p = 0. 003). The students from years 1 and 4 showed differences in opinion about the ‘roles and responsibility’. The year 4 students scored significantly higher than the year 1 students (F (4,804) = 4. 45; p = 0. 008). The detailed result is mentioned in Table 5. Interestingly, there was no difference in opinion by IEPS domains among the students based on the different year of study.

**Table 5:** RIPLS and IEPS domain scores for different year of study (mean and standard deviation).

**Domains**	**Year 1 Mean(SD)**	**Year 2 Mean(SD)**	**Year 3 Mean(SD)**	**Year 4 Mean(SD)**	**Year 5 Mean(SD)**	**F- ratio**
**RIPLS**	**73.57 (3.81)**	**74.86(3.27)**	**75.36 (3.74)**	**77.07 (3.60)**	**76.52 (1.42)**	
Teamwork and collaboration	39.46 (3.65)	38.76 (3.59)	38.91 (3.75)	39.16 (3.93)	38.57 (3.04)	F(4,804) = 4.45 p = 0.001
Negative professional identity	9.71 (3.45)	10.80 (2.24)	10.82 (3.31)	11.78 (2.73)	11.42 (1.99)	F(4,804) = 6.43 p = 0.003
Positive professional identity	16.20 (2.27)	16.59 (1.85)	16.83 (2.28)	17.13 (2.01)	17.21 (1.97)	F(4,804) = 1.98 p = 0.267
Roles and responsibility	8.20 (2.07)	8.71 (2.14)	8.80 (1.88)	9.00 (2.12)	9.32 (2.14)	F(4,804) = 4.45 p = 0.008
**IEPS**	**45.55 (5.45)**	**47.33 (5.87)**	**46.89 (6.27)**	**48.66 (6.01)**	**48.54 (5.61)**	
Competency and Autonomy	18.35 (2.06)	19.71 (2.94)	20.14 (2.14)	20.59 (1.85)	20.45 (2.19)	F(4,804) = 0.95 p = 0.407
Perceived need for Cooperation	7.06 (1.22)	7.48 (1.04)	7.75 (1.12)	8.10 (1.32)	7.48 (1.32)	F(4,804) = 1.08 p = 0.486
Perception of actual cooperation	20.14 (2.17)	20.14 (1.89)	19.00 (3.01)	19.97 (2.84)	20.61 (2.10)	F(4,804) = 0.89 p = 0.693

## Discussion

The positive effects of interprofessional practice in improved patient care have been described by many of the researchers worldwide. Patient-centric care has been shown to be important and can be facilitated by IPL in medical and healthcare-related educational programmes [19]. The degree of student preparedness in IPL predicts the likelihood that they may engage in interprofessional practice as future healthcare providers. The present results showed that the students from various health professional courses have been prepared to accept IPL as an important element in healthcare.

Students from different programmes appreciated the role of shared learning with other health professional courses in recognising and managing clinical problems during their studies [20, 21]. Tan et al., (2014) reported that shared learning experiences influence students’ perceptions of and attitudes to IPL and help them prepare for future collaborative practice [22]. Morison et al., (2004) reported that medical students appreciated shared learning sessions when learning about teamwork and the role of other healthcare professionals [23]. Our results are in line with this report. The respondents in our study concurred that their experience of shared learning can develop their skills in communication, especially when managing their patients as well as interacting with various healthcare providers. Improving communication skills is one of the major reasons for successful collaborative practice in healthcare [4]. The lowest score for the items ‘*I have to acquire much more knowledge and skill than other students’* and *‘I am not sure what my professional role will be’* indicated the students' poor understanding of their own roles and responsibilities. Understanding their own professional role in the healthcare team is of great importance [24] and lack of clarity about these roles may lead to them not working well with other health professionals. The complexity of intra- and inter- professional relationships may not be clearly understood by the students. Therefore, a common framework for the curricula for all healthcare profession students must be developed and a platform for interprofessional communication must be created during the early stages of their professional education [25]. Each healthcare professional should be able to identify and understand the unique differences between various disciplines in the healthcare workplace, yet function as a team [26].

Students mentioned that they will work well with individuals if they are from their own profession. Also, they are willing to share information and resources with other professionals. However, students disagreed that they could work closely with individuals from other professions and mentioned that they do not have good relations with people from those professions. This finding is consistent with earlier reports [27–29]. Understanding students' attitudes towards other professions will be a key consideration in the planning of interprofessional curricula. The students’ attitudes need to be developed at the entry level of the programme as, at that stage, they have their personal judgments and perceptions [30]. Therefore, IPL should be included in the early years of their professional education before they develop stereotypical views of other health professionals [31]. This is supported by an earlier study showing that ‘contact hypotheses’ help the group to modify their beliefs about IPL and can greatly influence their attitudes towards interprofessional practice [32]. Horsburgh et al., (2001) reported that undergraduate students believed that they needed to obtain more skills and knowledge than other allied healthcare providers [28]. This may then have a deeper impact on their attitudes towards interprofessional relationships and may have contributed to their attitudes towards IPL. Other factors such as the learning environment, as well as their open-mindedness towards cooperation with other professions will inevitably play a part in their perceptions and attitudes.

The mean scores for the RIPLS domains ‘teamwork and collaboration’, ‘positive professional identity’ and their ‘roles and responsibilities’ were not significantly different among students from different disciplines. This shows that students from all disciplines were ready for team working, encouraging positive professional relationship. In order to better understand other health professionals’ role in the healthcare team, every healthcare professional student must understand their own professional identity at the initial stage of their professional career. This would help them to better engage with their colleagues from other professions and enhance their ability to solve healthcare related problems together [33]. We found that medical students had significantly higher ‘negative professional identity’ scores compared to students from other disciplines. Similar results were also reported by other studies [27–29]. This finding may be due to the lack of exposure of medical students in managing multi-disciplinary healthcare team. Medical schools must focus on the curriculum to provide an opportunity for the students to learn and practise interprofessional collaboration in healthcare. Another significant finding from IEPS was the difference in ‘competency and autonomy’ domain between students from medical and other disciplines. Hawk et al., (2002) reported similar results [34]. The longer duration of the medical course and an increased exposure in the clinical setting might have influenced the medical students' perception of interprofessional practice [35]. Also, the medical students have more observation-based learning and regular clinical practice during their course work. However, the domains ‘perceived need for cooperation’ and ‘perception of actual cooperation’ did not reveal any significant differences between the students from different disciplines. Similar results were reported by Gooding et al., 2016 [36].

Analysis of the RIPLS data indicated that students’ attitudes towards IPL change across the years of study. Similarly, Lindqvist et al., reported that there is a significant relationship between the 'years of study’ and the RIPLS scores [30]. This is in agreement with our findings concerning the ‘team-work and collaboration’, ‘negative professional identity’ and ‘roles and responsibility’ domains of the RIPLS. The change in their thinking about the roles and responsibilities of each profession and their recognition of the need for team work may be due to their increased clinical exposure in later years, together the longer periods of higher order thinking associated with later stages of the course. The results also highlighted the change in perception with cohort seniority. The roles of the students have gradually changed across 1 to 5 years of the course, along with an increase in their responsibilities. This observation showed that learners’ attitudes can change with the amount of time they spend in their academic institution and practice site. This may also be enhanced by interactions with instructors/preceptors and peers at the clinical site [37]. There is a need for students from each profession to develop skills to practise within the multiprofessional healthcare team, in order to achieve a better patient outcome. Educators and policy makers should develop strategies to observe students’ behaviours and perceptions qualitatively [8].

## Limitations

The survey received responses from the majority of the students in the study population, with very few non-respondents. Non-response is a common problem in survey based studies. Nevertheless, the discipline-wise response rate for this survey is between 71–94% and the survey reached a minimum sample size required. As the percentage of non-responders was very small, we did not survey this group; non-response bias is highly unlikely to influence the results of the present study.

The cross-sectional and explorative design of this study should be taken into account when generalizing the data. Due to the design of this study and sampling from a single study site, the results may be a snapshot and may not be able to be extrapolated to other universities. Although the results from this study are reliable and robust, enabling the planning of educational strategies for interprofessional collaborative practice, a longitudinal study using all the universities in Malaysia is required to strengthen the findings.

## Conclusions

Attitudes towards and readiness for IPL showed significant differences among students of various healthcare professions, as well as their year of study. IPL should be incorporated in the curriculum of all healthcare professional programs; this may foster the development of skills to practise in a multidisciplinary healthcare setting as well as helping students to understand the roles of individuals from other professions. Future research should focus on specific factors that may have affected students’ attitudes to and readiness for IPL. Focus group discussions may be helpful to define how IPL occurs in each year of the study and how it influences the change in students’ perception of and attitudes towards IPL.

## Supporting Information

S1 File **Figure A. Questionnaire- Respondents’ demgraphic details.** Figure A shows the details of questions asked to collect respondents’ demography. **Figure B. The Readiness for Interprofessional Learning Scale (RIPLS).** Figure B shows the RIPLS scale used in the survey. **Figure C. The Interdisciplinary Education Perception Scale (IEPS).** Figure C shows the IEPS scale used in the survey.
(DOCX)

## References

[1] Mitchell P, Wynia M, Golden R, McNellis B, Okun S, Webb CE, et al Core principles & values of effective team-based healthcare. Institute of Medicine, 2014 [Online]. Available at: www.iom.edu/tbc.

[2] Barwell J, Arnold F, Berry H. How interprofessional learning improves care. Nurs Times. 2013;109:14–16. 23798160

[3] Wakefield MK. The Quality Chasm Series: Implications for Nursing In: Hughes RG, editor. Patient safety and quality: An evidence-based handbook for nurses. Rockville (MD): Agency for Healthcare Research and Quality (US); 2008 Apr Chapter 4. Available at: http://www.ncbi.nlm.nih.gov/books/NBK2677/21328776

[4] Bridges DR, Davidson RA, Odegard PS, Maki IV, Tomkowiak J. Interprofessional collaboration: three best practice models of interprofessional education. Med Educ Online. 2011;16. 10.3402/meo.v16i0.6035 PMC3081249 21519399

[5] Riva JJ, Lam JM, Stanford EC, Moore AE, Endicott AR, Krawchenko IE. Interprofessional education through shadowing experiences in multi-disciplinary clinical settings. Chiropr & Osteopat. 2010;18:31. 10.1186/1746-1340-18-31 PMC3003232 21126343

[6] Hood K, Cant R, Baulch J, Gilbee A, Leech M, Anderson A, et al Prior experience of interprofessional learning enhances undergraduate nursing and healthcare students' professional identity and attitudes to teamwork. Nurse Educ Pract. 2014;14:117–122. 10.1016/j.nepr.2013.07.013 23937910

[7] Sigalet E, Donnon T, Grant V. Undergraduate students' perceptions of and attitudes toward a simulation-based interprofessional curriculum: the KidSIM ATTITUDES questionnaire. Simul Healthc. 2012;7:353–358. 10.1097/SIH.0b013e318264499e 22902608

[8] Kingston R, Mari Kannan M, Suan Phaik K, Dinesh Kumar C, Ranjit De A, Hui Cing C et al Suitability of the RIPLS and IEPS for Discriminating Attitude Differences towards Interprofessional Education among Students of Healthcare Profession. Educ Res Int. 2016. Article ID 5946154.

[9] Judge M, Polifroni E, Maruca A, Hobson M, Leschak A, Zakewicz H. Evaluation of students' receptiveness and response to an interprofessional learning activity across healthcare disciplines: An approach toward team development in healthcare. International Journal of Nursing Sciences 2015;2:93–98.

[10] Gilbert J. Interprofessional education for collaborative, patient-centred practice. Can J Nurs Leadersh. 2005;18:32–36. 10.12927/cjnl.2005.17181 16045054

[11] O'Neill BJ, Wyness MA. Learning about interprofessional education: student voices. J Interprof Care 2004;18:198–200. 10.1080/13561820410001686954 15203678

[12] Buring SM, Bhushan A, Broeseker A, Conway S, Duncan-Hewitt W, Hansen L, et al Interprofessional education: definitions, student competencies, and guidelines for implementation. Am J Pharm Educ. 2009;73:59 19657492 10.5688/aj730459 PMC2720355

[13] Oandasan I, Reeves S. Key elements for interprofessional education. Part 1: The learner, the educator and the learning context. J Interprof Care 2005;19:21–38. 10.1080/13561820500083550 16096143

[14] World Health Organization. Framework for action on interprofessional education and collaborative practice 2010 WHO: Switzerland 2012. Available at: http://www.who.int/hrh/resources/framework_action/en/21174039

[15] Parsell G, Bligh J. The development of a questionnaire to assess the readiness of healthcare students for interprofessional learning (RIPLS). Med Educ. 1999;33:95–100. 10211258 10.1046/j.1365-2923.1999.00298.x

[16] Wilhelmsson M, Ponzer S, Dahlgren LO, Timpka T, Faresjo T. Are female students in general and nursing students more ready for teamwork and interprofessional collaboration in healthcare? BMC Med Educ. 2011;11:15 10.1186/1472-6920-11-15 21510872 PMC3110123

[17] Coster S, Norman I, Murrells T, Kitchen S, Meerabeau E, Sooboodoo E, et al Interprofessional attitudes amongst undergraduate students in the health professions: A longitudinal questionnaire survey. Int J Nurs Stud. 2008;45:1667–1681. 10.1016/j.ijnurstu.2008.02.008 18423644

[18] Horsburgh M, Lamdin R, Williamson E. Multiprofessional learning: the attitudes of medical, nursing and pharmacy students to shared learning. Med Educ. 2001;35:876–883. 11555226 10.1046/j.1365-2923.2001.00959.x

[19] Thistlethwaite J. Interprofessional education: a review of context, learning and the research agenda. Med Educ. 2011;46:58–70. 10.1111/j.1365-2923.2011.04143.x 22150197

[20] Williams B, Boyle M, Brightwell R, McCall M, McMullen P, Munro G, et al A cross-sectional study of paramedics' readiness for interprofessional learning and cooperation: Results from five universities. Nurse Educ Today 2013;33:1369–1375. 10.1016/j.nedt.2012.06.021 22818954

[21] Holthaus V, Sergakis G, Rohrig L, Wilcox J, Thomas E, Mcclerking C, et al The impact of interprofessional simulation on dietetic student perception of communication, decision making, roles, and self-efficacy. Top Clin Nutr. 2015;30:127–142.

[22] Tan CE, Jaffar A, Tong S-F, Hamzah MS, Mohamad N. Comprehensive healthcare module: medical and pharmacy students’ shared learning experiences. Med Educ Online 2014;19. 10.3402/meo.v19.25605 PMC4202196 25327980

[23] Morison S, Boohan M, Moutray M, Jenkins J. Developing pre-qualification inter-professional education for nursing and medical students: sampling student attitudes to guide development. Nurse Educ Pract. 2004;4:20–29. 10.1016/S1471-5953(03)00015-5 19038133

[24] Lister L. Role training for interdisciplinary health teams. Health Soc Work. 1982;7:19–25. 7076046 10.1093/hsw/7.1.19

[25] Hughes RG. Nurses at the “Sharp End” of Patient Care In: Hughes RG, editor. Patient Safety and Quality: An Evidence-Based Handbook for Nurses. Rockville (MD): Agency for Healthcare Research and Quality (US); 2008 4 Chapter 2. Available from: http://www.ncbi.nlm.nih.gov/books/NBK2672/21328752

[26] Insalaco D, Ozkurt E, Santiago D. Attitudes and knowledge of students in the allied health professions toward their future professional team members. J Allied Health 2006;35:142–146 17036668

[27] Aziz Z, Teck LC, Yen PY. The attitudes of medical, nursing and pharmacy students to inter-professional learning. Procedia—Social and Behavioral Sciences 2011;29:639–645.

[28] Horsburgh M, Lamdin R, Williamson E. Multiprofessional learning: the attitudes of medical, nursing and pharmacy students to shared learning. Med Educ. 2001;35:876–883. 11555226 10.1046/j.1365-2923.2001.00959.x

[29] Reid R, Bruce D, Allstaff K, Mclernon D. Validating the Readiness for Interprofessional Learning Scale (RIPLS) in the postgraduate context: are healthcare professionals ready for IPL? Med Educ. 2006;40:415–422. 10.1111/j.1365-2929.2006.02442.x 16635120

[30] Lindqvist S, Duncan A, Shepstone L, Watts F, Pearce S. Case-based learning in cross-professional groups–the development of a pre-registration interprofessional learning programme. J Interprof Care 2005;19:509–520. 10.1080/13561820500126854 16308173

[31] Carpenter J, Hewstone M. Shared learning for doctors and social workers: Evaluation of a programme. Br J Soc Work 1996;26:239–257.

[32] Hean S, Dickinson C. The contact hypothesis: An exploration of its further potential in interprofessional education. J Interprof Care 2005;19:480–491. 10.1080/13561820500215202 16308171

[33] Harden RM. "AMEE Guide No. 12: Multiprofessional Education: Part 2—Effective Multiprofessional Education: A Three-dimensional Perspective." Medical Teacher 1998;20:409–416.

[34] Hawk C, Buckwalter K, Byrd L, Cigelman S, Dorfman L, Ferguson K. Health Professions Studentsʼ Perceptions of Interprofessional Relationships. Acad Med. 2002;77:354–357. 11953306 10.1097/00001888-200204000-00020

[35] Page RL, Hume AL, Trujillo JM, Leader WG, Vardeny O, Neuhauser MM, et al Interprofessional education: principles and application. A framework for clinical pharmacy. Pharmacotherapy. 2009;29:145e–164e.

[36] Gooding HC, Ziniel S, Touloumtzis C, Pitts S, Goncalves A, Emans J, et al Case-Based Teaching for Interprofessional Postgraduate Trainees in Adolescent Health. J Adolesc Health 2016;58:567–572. 10.1016/j.jadohealth.2016.01.011 27107910

[37] Terenzini P, Pascarella E. Living with myths: Undergraduate education in America. Change. 1994;26:28–32.

