# Supplementary material for: Attitudes and Readiness of Students of Healthcare Professions towards Interprofessional Learning
Source: PLoS One. 2017 Jan 6;12(1):e0168863. doi: 10.1371/journal.pone.0168863 (PMC5217964; doi:10.1371/journal.pone.0168863)
Supplement: S1 File — Figure A. Questionnaire- Respondents’ demgraphic details. Figure A shows the details of questions asked to collect respondents’ demography. Figure B. The Readiness for Interprofessional Learning Scale (RIPLS). Figure B shows the RIPLS scale used in the survey. Figure C. The Interdisciplinary Education Perception Scale (IEPS). Figure C shows the IEPS scale used in the survey. (DOCX) [file pone.0168863.s001.docx]

**Figure A Part 1 of Questionnaire-demgraphic details**

**
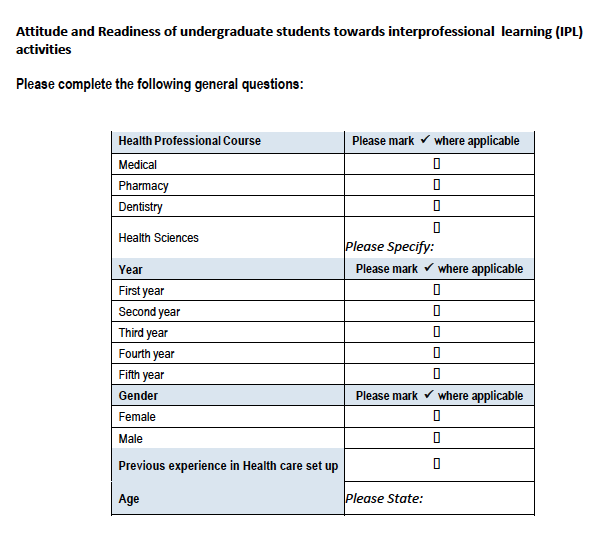
**

**Figure B The Readiness for Interprofessional Learning Scale (RIPLS)**

**
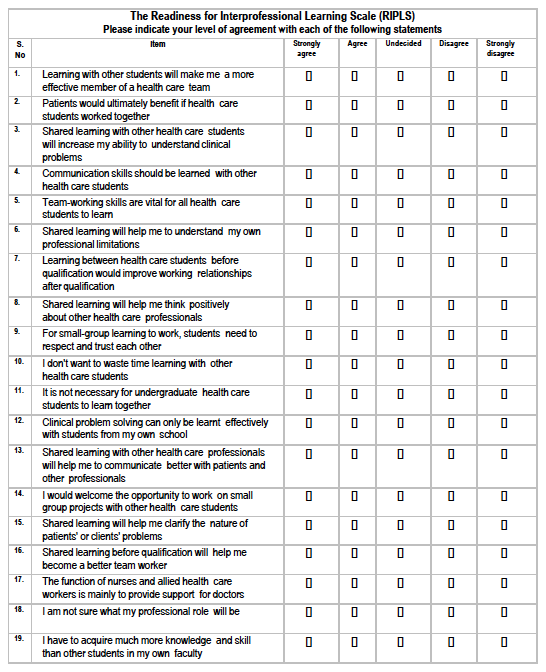
**

**Figure C The Interdisciplinary Education Perception Scale (IEPS)**

**
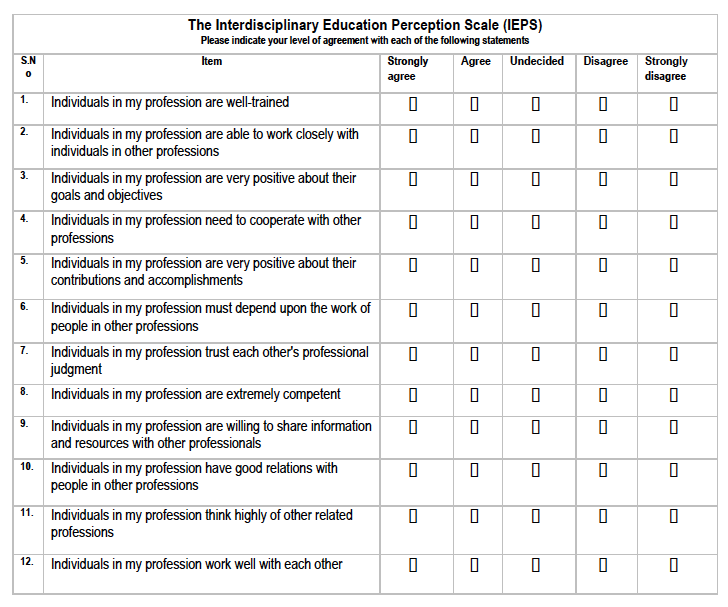
**
